# Presumptive primary intraocular lymphoma presented as an intraocular mass involving the optic nerve head

**DOI:** 10.1007/s12348-011-0045-7

**Published:** 2011-11-10

**Authors:** Alireza Hedayatfar, Soon phaik Chee

**Affiliations:** 1 Ocular Inflammation and Immunology Department, Singapore National Eye Centre, 11 Third Hospital Avenue, Singapore, 168751 Singapore; 2 Noor Eye Hospital, Tehran, Iran; 3 National University of Singapore, Singapore, Singapore; 4 Singapore Eye Research Institute, Singapore, Singapore

## Introduction

Primary intraocular lymphoma (PIOL) is a subset of primary central nervous system (CNS) lymphoma which initially presents in the eye with or without simultaneous CNS involvement (1). It is considered a variant of extra-nodal, non-Hodgkin lymphoma that is usually of an aggressive diffuse large B cell type (1–3). Diagnosis of PIOL requires histopathologic evidence of malignant lymphoma cells in brain biopsy, vitreous or CSF specimen (1, 2). Cytological examination is the mainstay method to detect lymphomatous cells (3, 4). Other ancillary tests like flow cytometry, immunohistochemistry staining, cytokine analysis, and gene rearrangement studies also aid in the diagnosis and further classification of PIOL (1, 4).

Optic nerve and optic disc involvement may occur in the PIOL (4, 5). Here, we report a case with an unusual presentation of this malignancy presented as an intraocular mass with involvement of the optic nerve head.

## Case report

**Fig. 1:**
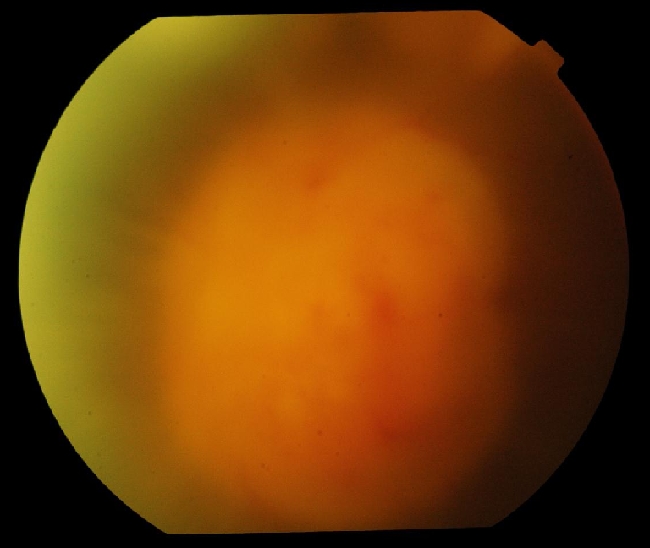
Fundus photograph shows an intraocular mass over the optic disc with overlying vitreous haze

A 52-year-old healthy female presented with a 2-month history of sudden and progressive painless visual impairment in her left eye. Careful review of history did not reveal additional clues except that she had been having mild frontal headache for 2 years. On examination, her visual acuity was 6/6 and light perception in right and left eyes, respectively. There was a grade 3 left relative afferent pupillary defect. The left anterior chamber had a few non-granulomatous keratic precipitates and +1 cells. Dilated fundal examination revealed a large yellow–whitish intraocular mass above the optic disc with overlying vitritis (Fig. 1). B-scan ultrasound study confirmed the solid nature of the mass and its exact location of adherence to optic disc.

**Fig. 2:**
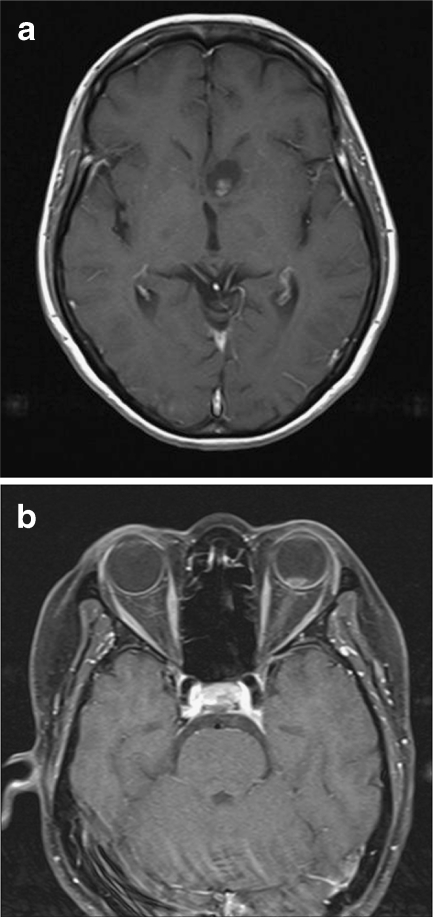
Gadolinium-enhanced brain and orbital magnetic resonance images show a cystic mass with a solid internal enhancing component in left caudate nucleus (**a**) and an enhanced solid mass in the left eye over the optic nerve head (**b**)

Systemic examination and investigation, including a complete blood count, erythrocyte sedimentation rate, C-reactive protein, angiotensin-converting enzyme titer, urine analysis, and chest X-ray were unremarkable. Skin tuberculin test and blood serology for syphilis, borreliosis , toxoplasmosis, and human immunodeficiency virus were negative. Smear and culture of vitreous specimen did not show the evidence of bacteria or fungi. Vitreous tap was done but cytology did not reveal the presence of malignant cells. Interlukin-10 level and IgH gene rearrangement studies were inconclusive for lymphoma. At this time, a brain and orbital magnetic resonance imaging (MRI) revealed a cystic mass with an enhanced solid component in left caudate nucleus. There was also a smaller enhanced solid mass in the left eye over the optic nerve head (Fig. 2a, b).

**Fig. 3:**
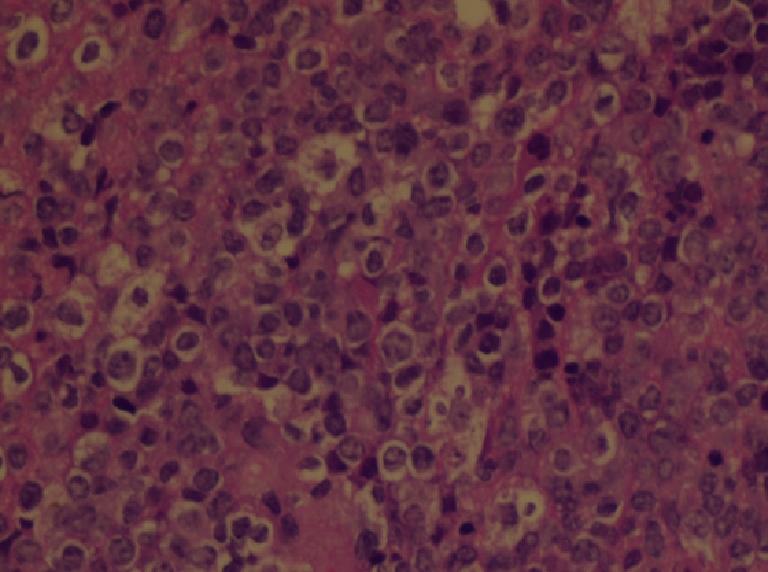
Histopathologic section of caudate nucleus mass (H&E × 400): Sheets of packed, pleomorphic large cells with high nucleus to cytoplasm (N/C) ratio, consistent with a diffuse large cell lymphoma

Navigation-guided (Stereotactic) biopsy of caudate nucleus lesion revealed a hypercellular lesion, consisting of sheets of large cells with pleomorphic vesicular nuclei and brisk mitoses. The histopathologic and immunohistochemistry features were consistent with the diagnosis of a large B cell lymphoma (Fig. 3).

**Fig. 4:**
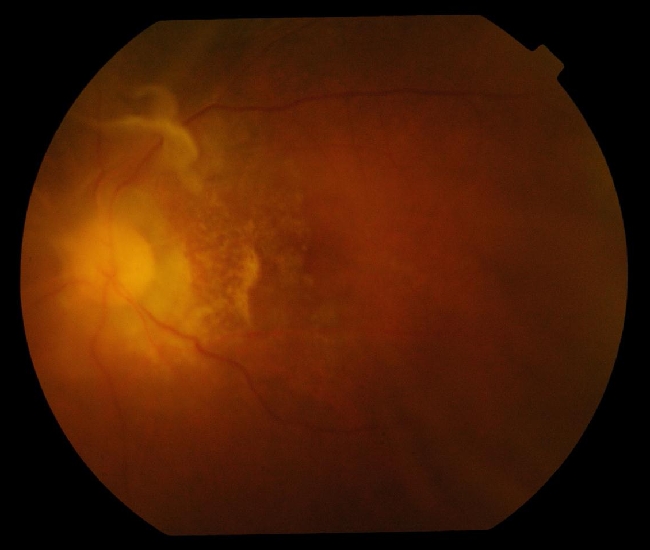
Fundus photograph of the same patient after chemotherapy: pale optic disc together with prepapillary atrophic lesions. Note the disappearance of the mass and vitreous haziness

Systemic examination and further extensive investigations did not show involvement of other sites. She was diagnosed with the primary intraocular-CNS lymphoma and was treated with the De Angelis chemotherapy protocol without adjunctive radiotherapy. One month later, the brain MRI revealed a remarkable decrease in the size of intracranial tumor, and fundus examination showed significant decrease in the vitreous haze with disappearance of the mass, but the visual acuity did not improve, and she remained with a pale optic disc and some peripapillary atrophic lesions (Fig. 4).

## Discussion

Primary intraocular lymphoma is considered one of the most elusive ocular masquerade syndromes. The diagnosis is frequently delayed due to misdiagnosis and false-negative biopsy reports which may be a result of small sample size (inadequate sample, too few vitreous cells, and sampling from out of the lesion), improper handling of specimen (unsuitable transferring media, delay in transferring or processing the specimen, and high temperature), or prior steroid therapy (1, 3). To lessen the false-negative results, our patient was instructed to stop oral steroids for 2 weeks before vitreous tap. Pathologist was first alerted, and then specimen was obtained fresh and transported immediately to the cytologist for prompt processing. In spite all measures, the initial vitreous specimen was negative for malignant cells. In our case, the vitritis was limited to the posterior vitreous (just around the mass) which may result in low diagnostic yield by vitreous tap. There would probably have been a higher chance to obtain the malignant cells had a diagnostic vitrectomy been performed. The dramatic clinical response to systemic chemotherapy which was manifested by the reduction of the vitreous cells and the size of intraocular mass convinced us that nature of both intraocular and intracranial lesions must be the same.

In our patient, the optic disc was infiltrated by malignant cells. This was revealed by close vicinity between the intraocular mass and optic disc in B-scan and magnetic resonance images. The profound loss of vision and significant relative afferent pupillary defect early in the course of the disease further support this assumption. However, due to lack of confirmatory vitreous biopsy, we considered the nature of the mass presumptive.

Although optic nerve and optic disc infiltration have been reported in primary intraocular lymphoma (4, 5), this case represents an unusual presentation of presumptive intraocular lymphoma featuring as an intraocular mass with involvement of optic nerve head that to our knowledge has not been reported yet.
